# Exploiting the Circadian Clock for Improved Cancer Therapy: Perspective From a Cell Biologist

**DOI:** 10.3389/fgene.2019.01210

**Published:** 2019-12-11

**Authors:** Tia Tyrsett Kuo, Andreas G. Ladurner

**Affiliations:** ^1^Biomedical Center Munich, Faculty of Medicine, Ludwig-Maximilians-Universität München, Planegg-Martinsried, Germany; ^2^Max Planck Institute of Biochemistry, International Max Planck Research School for Molecular and Cellular Life Sciences, Martinsried, Germany; ^3^Center for Integrated Protein Science Munich (CIPSM), Ludwig-Maximilians-Universität München, Munich, Germany; ^4^Munich Cluster for Systems Neurology (SyNergy), Ludwig-Maximilians-Universität München, Munich, Germany

**Keywords:** chronotherapeutic, circadian rhythm, chemotherapy, cancer, precision (stratified) medicine

## Introduction

Since the discovery of the biological clock, the concept of treating cancer according to biological rhythms, here termed cancer chronotherapy, has rapidly evolved. Its fundamental aim is to improve the efficacy of drugs and to minimize adverse effects by administering chemotherapeutic drugs at the appropriate time-of-day. In the last two decades, several experimental and clinical studies have reported positive associations between the circadian clock and drug response in cancer patients. However, the lack of mechanistic insights into critical, deterministic clock-controlled genetic, and metabolic variations between and within individual cancer patients continue to cast a shadow on the potential benefits cancer chronotherapy may provide. Here, we provide first a simplified overview on our biological clocks and how our life-style induces complex biochemical reactions and genetic interactions. Next, we summarize how these reactions directly and indirectly modulate the effectiveness and toxicity of oncological drug treatments. Since cytotoxic chemotherapy represents the most common and affordable of cancer treatments, a case should be made that we need to ensure these treatments are used in the best possible manner. Thus, we list current challenges and future directions toward that goal.

## Interview With Professor Francis Albert Lévi, Clinical Professor of Biomedicine At the University of Warwick Medical School, EMBO | EMBL Symposium: Biological Oscillators: Design, Mechanism, Function

Professor Francis Albert Lévi is Clinical Professor of Biomedicine – Medical Oncology at the University of Warwick Medical School and Honorary Consultant at the Cancer Center, University Hospital Birmingham Queen Elisabeth. His team aims to streamline basic and clinical research on biological clocks, drugs, and diseases to promote the integration of chronotherapy into drug development and daily medical practice. The ultimate goal of Prof. Francis Lévi and his team is to predict and prevent diseases through the fine-tuning of our biological rhythms.

## What Is Currently the Main Challenge in Cancer Chronotherapy?

“The scientific and technological challenges are to adjust chronotherapy to the circadian clock in both healthy and tumor tissues of cancer patients. To do this, we need to stratify cancer patients according to how their circadian clocks are working, and to identify the optimal timing for the delivery of chronotherapy accordingly. This means that we would need to develop five to ten Phase I-II and III clinical trials with strong translational components and have them funded through long-term investment strategies. In parallel, we need Regulatory Agencies to make the recording of time, day, and month of treatments and sample collections a mandatory information during drug trials, and convince pharmaceutical companies that drug development could greatly benefit from considering timing issues during preclinical and early clinical testing. This could help us obtain the clinical data that we need in order to evaluate the potential benefit of therapeutic strategies.”

## How Could Basic Research Help Advance the Field?

“Generally, basic chronopharmacology research involves a standardized, systematic approach when studying physiology and pharmacology in animals or cultured cells. This allows us to generate a substantial amount of quantitative information on time-dependent drug pharmacology, efficacy, and toxicity. We need mathematical approaches to systematically map cancer chronopharmacology mechanisms in cellular models with synchronized clocks. Next, we need the ability to adjust these models established in rodent models in healthy volunteers and in cancer patients. Thus, a systems chronopharmacology approach going from cells to whole organisms would appear to be the currently best available option for modern cancer chronotherapeutics. In addition, novel approaches aiming to enhance the robustness of the circadian clocks in the host or in the cancer also represent a way forward, as illustrated by the recent findings of Dr. Panda and his team (Mure et al., 2018). However, as suggested in their studies, primates and mice share very few common genes of which display rhythmical expression. This finding highlights the diversity of the molecular clock across species. Thus, the design and evaluation of chrono-modulatory treatments in animal models calls for additional attention.”

## What Is the Outlook on Cancer Chronotherapy?

“With our constantly expanding mechanistic understanding of the molecular clock and its dynamic interaction with many metabolites, with the cell cycle and with cell survival pathways, as well as the refinement of mathematical models applied to systems-level data, I believe we will soon be able to change the landscape of chronotherapy. I look forward to seeing you, along with all other young scientists, working further and deeper on the biological clock in order to achieve this goal.”

## Rationale

Despite immense advances in cancer research and the development of novel therapies in oncology over the past decades, cancer remains one of the leading causes of death worldwide. According to the World Health Organization’s (WHO) estimates of cancer incidence and mortality of all ages and both sexes presented for 2012, there were c.a. 1.8 million new cases and 1.6 million deaths of lung cancer alone. Therapeutic treatment options for many of these cancers are limiting, despite of intense research activities and many technological and biological innovations. Indeed, from a standpoint of pharmaceutical development, recent analysis on the development of novel oncologic drugs for breast, colorectal, and nonsmall cell lung cancer show very high attrition rates. Between 1979 and 2014 (Nixon et al., 2017), attrition rate for new cancer therapies has been significantly high. Over 80% for drug classes including cytotoxic chemotherapeutic reagents and cancer immunomodulators failed to advance to later stages in clinical trial. According to this analysis, oncological drugs are more likely to fail in late stage clinical trials (transition from Phase II to III) compared to other drugs. The authors concluded that adjusting and refining the design of early stage clinical trials may aid in the successful selection of drugs in later and more expensive stages of drug development (Nixon et al., 2017).

An additional, important aspect – and possibly the real, “biological” crux of the matter at hand – is the distinct variation in drug responses between individual patients. This highlights the urgency and a need to understand the variables that exist between individuals in order to improve the current status of cancer treatment. Such knowledge could clearly benefit the testing and selection of potential drugs for further development and clinical testing. From this standpoint, the distinct genetic and epigenetic make-up of individuals contributes greatly to individual variation. Increasingly, we will therefore likely see basket trials in oncology, for example, where patients are stratified based on genetic/genomic markers, such as the absence of a particular tumor suppressor gene, for example, rather than the etiology of the tumor itself. Moreover, emerging evidence in chronobiology strongly supports the intertwining relationship between our cyclic environment, behavior, and physiology, as well as the influence on the biochemical and genetic activity of our tissues and organs.

The question then is how we can exploit our fundamental knowledge of environmental and metabolic cycles to improve cancer treatment? While many factors come into play, this brief perspective focuses on how an appreciation for circadian biology might improve on the clinical use of global care standards in cancer chemotherapy.

## Laying the Foundation for Cancer Chronotherapy

Over half a century ago, Dr. Mauricio Garcia-Sainz and Dr. Franz Halberg identified, through computational analyses, a 24-h mitotic rhythmicity in human mammary but not squamous and basal cell cancer biopsy samples obtained before or after radiotherapy (Garcia-Sainz and Halberg, 1966). They were able to distinguish the variance as rhythmicity <20 h and observed a population reduction of such ultradian rhythmicity in samples obtained after radiotherapy. This discovery established chronopathology and opened up the conceptual possibility for the development of chrono-modulated therapies. However, the potential of this information did not fully blossom until the identification of the first clock gene by Michael W. Young, Michael Rosbash, and Jeffrey C. Hall in the 1990’s. Since then, Prof. Francis Albert Lévi, a clinician and scientist, his team, as well as others in the field have been actively researching and promoting the concept of targeting the circadian clock to improve current cancer treatments* (see a short interview with Prof. Lévi on page XX of this perspective). However, to date, cancer chronotherapy faces numerous challenges, some of which we will discuss in this article. In the following sections, we will provide a simplified summary of the circadian clock, how circadian regulatory network extends into chemotherapeutics, and summarize up-to-date implications of chronobiology in cancer therapy *https://warwick.ac.uk/fac/sci/med/research/biomedical/labs/chronotherapy/about/.

## Life Evolved in Sync With the Environment

Due to the Earth’s rotation, virtually all life has adapted and evolved an approximately 24-h biological rhythmicity in order to anticipate periodic changes in our environment, such as light, temperature, and food availability. This behavioral and physiological rhythmicity driven by natural sunlight is termed the circadian clock (*circa diem*). One tenable theory is that organisms evolved to store and utilize energy in synchrony with its environments. Thus, most fundamental biological processes such as cell metabolism and growth are thought to follow a daily circa 24-h oscillation [for a review, see (Takahashi, 2016)]. However, a recent metabolomic analysis has elegantly demonstrated the existence of other (non-24-h) rhythmicity and periodicity as well (Krishnaiah et al., 2017). Overall, cellular processes are thus greatly subjected to cyclic environmental cues, responses, and behaviors.

## Light Entrains the Central Clock

In animals, the eye possesses the ability to sense light. Upon exposure to light, complex signals are sent through the retinohypothalamic tract and subsequently processed in the suprachiasmatic nucleus (SCN). In the SCN, a relatively small population of specialized cells regulates the synthesis of the hormone melatonin by the pineal gland. The result of fluctuating melatonin levels, in turn, triggers the “resetting” of peripheral clocks, such as the liver and kidneys. These seemingly simple neurochemical and hormonal processes drive the most overt biological rhythmicity, the sleep-wake cycle, and is regarded as the central clock [for a review, see (Korf and von Gall, 2013)]. However, the molecular mechanism of how melatonin and likely other hormones synchronize peripheral tissues remains unclear.

## Nutrients Regulate Peripheral Clocks

While natural cyclic exposure to light remains the key driver of our biological clocks, nutrients have emerged as another key factor in the synchrony of peripheral clocks. Liver, regarded as the metabolic hub, has been extensively characterized as a circadian organ (Koike et al., 2012). Transcriptomic and proteomic analyses on mice liver demonstrate the diurnal transcriptional and post-translational regulation of discrete cellular processes, which range from glucose and fatty acid metabolism to the cell cycle and autophagy (Robles et al., 2016; Wang et al., 2018). In agreement, several clinical studies have reported circadian rhythmicity in glucose, lipid and energy metabolism, such as insulin sensitivity and secretion, cholesterol synthesis, and fat oxidation (Poggiogalle et al., 2018). These findings further strengthen the concept of multiple cellular processes being under the direct and indirect influence of our environment, nutrition, behavior, and metabolism.

## The Molecular Clock Drives Transcriptional Rhythmicity

At the cellular level, neurochemical and hormonal cues induced by light and nutrients prompt the resetting of the molecular clock. However, the molecular clock is governed by several interlocking transcription and translation feedback loops (TTFLs), which proceeds for a long time even in the absence of external cues. A recent, groundbreaking circadian transcriptomics study in the olive baboon (*Papio anubis*) revealed over 80% of protein-coding genes displayed a daily oscillation pattern in more than 60 examined tissues (Mure et al., 2018). The majority of cycling genes were mainly ubiquitously expressed genes, which are often involved in fundamental cellular processes, such as circadian rhythmicity, DNA replication, DNA repair, amino-sugar metabolism, and oxidative phosphorylation (Mure et al., 2018).

To conclude, the biochemical and genetic response of a metazoan organism is influenced not only by our predetermined genetic and epigenetic make-up; but also by a plethora of ever-changing external stimuli, including light and nutrients. Thus, in addition to well-studied genetic (Kandoth et al., 2013) and metabolic alterations (DeNicola and Cantley, 2015) in cancer, we must now take into account both “clock alterations” and clock regulatory genetic and metabolic networks. Only in combination, will we potentially succeed in profiling cancer subtypes and strategizing treatments. In the following section, we will present our case on cancer chronotherapy and up-to-date findings on the relationship between the clock, cancer, and chemotherapeutics.

## Circadian Clock Function Influences Cancer and Vice-Versa

Several recent findings indicate that there is a correlation between the circadian clock and the etiology of cancer. One bioinformatic study reported that clock genes in human tumor samples have altered gene expression profiles in comparison to normal, adjacent tissues (Ye et al., 2018). To no surprise, they also found a correlation between clock gene expression and anticancer drug sensitivity in cancer cell lines (Ye et al., 2018). To support this, a number of experimental and clinical studies have reported a negative correlation between clock disruption during and/or after treatment with survival rate in cancer animal models and patients (Sephton et al., 2013; Lévi et al., 2014; Papagiannakopoulos et al., 2016). These findings indicate a dysfunctional or functional clock may serve as an indicator for the outcome of cancer progression and treatment response. However, circadian modulated anticancer drug response and the potential effect of our environment and behavior on cancer development lack well-defined molecular insights necessary to comprehend and exploit the clock for common cancer treatments. In the following paragraphs, we summarize state-of-the-art findings in explaining how the circadian clock may improve the current status of cancer chemotherapy.

## Cytotoxic Chemotherapy as the Global Standard of Care

There are various approaches targeted at treating cancer. Notably, in recent years, immunotherapeutic approaches have shown great promise and interest in the research and clinical community. Nevertheless, conventional chemotherapy, regardless whether administered as induction, combined, consolidation, or through other therapeutic strategies, remains one of the most broadly used and affordable treatments following surgical resection of tumor tissue. Despite current knowledge on how, for example, platinum-based and similar chemotherapeutic drugs function, debilitating adverse effects resulting from severe damage to normal and healthy tissues limits the dosing of drugs and treatment duration (Tacar et al., 2012; Dasari and Bernard Tchounwou, 2014). Using chemotherapeutic drugs at suboptimal doses and treatment duration, in turn, provides space for the cancer to develop drug resistance. Cancer therapy using chemotherapeutic agents thus persistently faces the major challenge of relapse. In the following, we summarize how the clock regulates the DNA damage repair machinery targeted by cytotoxic chemotherapeutical drugs, drug metabolism, and potentially drug resistance in cancer.

## The Molecular Clock Regulates the DNA Repair Machinery

Conventional chemotherapy targets cells with enhanced proliferation rates and/or an impaired DNA repair machinery to achieve elimination of cancerous cells (Corrie, 2008). The circadian clock regulates these key hallmarks of cancer of which includes cell proliferation, metabolism, and genome stability [for extensive reviews, see (Sancar et al., 2010; Masri et al., 2013; Panda, 2016)]. For example, cisplatin, a widely used and well-established chemotherapeutic drug in the treatment of numerous human cancers, exerts its function by crosslinking purine bases on DNA, causing bulky lesions. These bulky lesions interfere with DNA replication and overwhelm the DNA repair machinery, which generally results in cell death (for a review, see (Dasari & Bernard Tchounwou, 2014)). These bulky DNA adducts are typically sensed and resolved by the nucleotide excision repair (NER) pathway. The activity of one particular key DNA repair protein, xeroderma pigmentosum A (XPA), was shown to be under direct circadian control *in vivo* (Kang et al., 2009; Kang et al., 2010). This knowledge strengthens the notion of timed drug (such as cisplatin) administration in accordance to the circadian “trough” of the targeted cell process (in this case, NER repair machinery) to increase drug efficacy.

## Drug Resistance Emerges Out of Cell Mechanisms Under Circadian Control

Cancer is a highly heterogeneous disease, rendering it a challenge to target and treat, as the genome rapidly evolves and resistance mechanisms arise albeit the simplicity of “timely” targeting. There are several proposed mechanisms of resistance to chemotherapy. Beside suboptimal drug dosage and treatment duration, this includes changes in the cellular uptake and efflux of the chemotherapeutic agent, increased drug metabolism and elimination, as well as enhanced DNA repair machineries, mechanisms, which suppress tumor cell death (Mansoori et al., 2017). These resistance mechanisms emerge from processes that are controlled by the circadian clock (Sancar et al., 2010; Dallmann et al., 2014; Dallmann et al., 2016). Thus far, clinical studies have shown that several adverse effects experienced by cancer patients taking cisplatin-based chemotherapy decreases when cisplatin is applied in a chrono-modulated context (Li et al., 2015; Zhang et al., 2017). The results and outcomes of these studies indicate that the proper timing of drug administration reduces cisplatin-induced adverse effects in patients. Chronotherapy may thus potentially enable substantial increases in the maximally tolerated dose. This raises the possibility that more effective toxicity to the tumor cells could be achieved, providing potentially a higher selectivity relative to the toxic effect of chemotherapy on normal cells.

In sum, the clock regulates the fundamental cell processes, which are often the targeted machinery of cytotoxic anticancer drugs. The clock also modulates the absorption, metabolism, and elimination of these drugs. In combination, chrono-modulated drug administration provides the potential to optimize the dosage of drugs and duration of treatments to efficiently eliminate highly proliferative cancerous cells while reducing limiting adverse effects to circumvent drug resistance. The potential for chronotherapy thus arises out of the physiological link between the cytotoxic cancer agents and molecular processes that respond to these agents on an organismic and cellular level.

## Current Challenges and Future Directions in Cancer Chronotherapy

Several studies report clear changes in the effectiveness of cancer chronotherapy. However, significant interpatient variability persistently poses a major challenge for cancer chronotherapy to gain statistical power (Ballesta et al., 2017). Giacchetti and colleagues conducted a comparative meta-analysis on conventional and chrono-modulated chemotherapeutic treatments in 842 patients with metastatic colorectal cancer from three international randomized trials (Giacchetti et al., 2012). They found improved tumor response rate and overall survival, as compared to conventional chemotherapy in male patients by +3.3 months. Unexpectedly, the overall survival of female patients decreased by 1.8 months (Giacchetti et al., 2012). One pilot study involving 55 healthy people and 12 cancer patients found significant sex- and age-related variations in the coordination of individual circadian clocks as well (Komarzynski et al., 2018). These critical findings underscore the relevance of sex and age for cancer chronotherapy and require further investigations on their respective impacts for the scheduling of chronotherapy. Such variations likely stem from and result in the challenges listed below.

There are at least three challenges faced by cancer chronotherapy. These include:

1. There is insufficient mechanistic insight at the level of basic research of how cancer and chemotherapeutic drugs affect the molecular clock and vice versa. This hampers the design of appropriate chrono-modulated clinical trials.
  In the last decade, tremendous effort has been put into deciphering the structure, function, and regulatory network of the molecular clock and its core components. While the field is rapidly gaining new insights on these fundamental biological questions, we have yet directly addressed the impact of the clock and metabolic environment on cytotoxic drug sensitivity in a systematic manner. Thus, in our opinion, the field requires a systematic high-throughput cell (organelle)-based screening capability on the circadian-dependent effects of chemotherapeutic cytotoxicity in both model cancer and primary cell lines. This would help confer novel mechanistic information. Ultimately, such information could provide valuable data to generate unique combinatorial (cancer, drug, and clock) profiles which may serve as guidelines clinical trial designs.
2. In clinical research, large variations among patients’ clocks can alter the statistical power of trials comparing chronotherapy to conventional treatment in patients with varying chronotypes (circadian clock).
  Cancer profiling is under constant refinement from tumor localization, cell morphology, and cell surface biomarkers to genetic and epigenetic aberrations. We now must also acknowledge that the variation extends to our biological clocks. The design of chrono-modulated chemotherapy may benefit immensely from compulsory documentation of patient routines including sleep/wake cycles, activity, metabolic profile, and drug administration in all related drug trials. While this is of course a great challenge in terms of data gathering and analysis, the European Medicines Agency (EMA) and its nearing release of the EMA Clinical Trial Information System (CTIS) with uniformed regulations and absolute transparency will aid us in moving towards an improved era of personalized and precise medicine tailored to individual chronotypes.
  Making chronotyping simple and easy Some wear it for fashion; some wear it for health. Regardless, smart watches, apps (Gill and Panda, 2015, https://mycircadianclock.org/) or other digital devices have the ability of recording valuable information of our cyclic behavior, such as when we sleep, how we sleep, and when and how we eat and exercise. By combining a given patient’s “digital” chrono-data with “biological” chrono-data, such as biopsies and/or blood samples, clinicians could then start to successfully chronotype individual patients and strategize cancer treatments accordingly. Applying such routines to clinical trials could help stratify patients and the results of the effectiveness of particular pharmacological therapies.
3. Pharmaceutical companies should be incentivized to invest in (re-)examining the time-dependent efficacy and toxicity of chemotherapeutic drugs, or to design clock-based drugs.

As suggested earlier (Nixon et al., 2017), the adjustment and refinement of early stage clinical trial design could permit a higher success rate in anticancer drug development. To put this into perspective, it costs on average a striking $2.6 billion USD to develop a drug (DiMasi et al., 2016). Considering the attrition rate of 80%, chronotherapeutic approaches could lead to the development of effective new anti-cancer drugs or improved dosing regimens. Pharmaceutical companies should take into account the effect of the clock and recognize the potential in re-examining drugs to increase not only the therapeutic value of approved drugs, but also re-evaluate new chemical entities.

**Figure 1:**
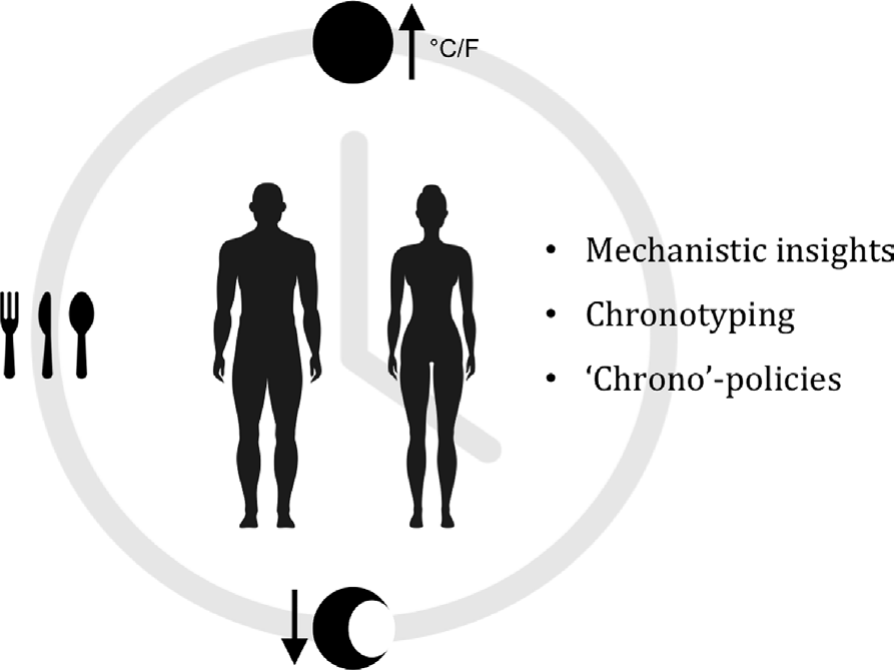
Exploiting the circadian clock for improved cancer therapy. We are exposed to ever-changing light, temperatures, and nutrients on a daily basis. In response to these Cyclic Stimuli, our bodies modulate a wide range of physiological and cellular processes in an oscillatory manner (Chronobiology). Since chronobiology is a complex physiological phenomenon, it is currently not greatly considered as a factor in the research and development of novel anticancer drugs, nor in clinical trials. Nonetheless, recent studies have reported correlations between the circadian clock, cancer and therapy response. Giacchetti et al., 2012; Sephton et al., 2013; Lévi et al., 2014; Papagiannakopoulos et al., 2016; Ye et al., 2018. We suggest that an in-depth understanding of circadian mechanisms from single tissue types to entire systems, could potentially allow us to stratify individual patients not only by genomic and genetic aberrations in their tumors, but also by clock functions (Chronotype). this could be of great benefit and improve cancer therapies.

In conclusion, these interlinking challenges require collaborative efforts between basic science and medicine, clinicians, regulatory authorities, and pharmaceutical companies in order to obtain mechanistic insights and clinical statistics that would help shed light on how a given cancer affects the clock and vice versa, how a given oncotherapy affects the clock, and what other factors, such as our lifestyle and environment (personal chronotypes), can affect the clock and treatment responses. The landscape of chronotherapy is gradually maturing with the application of *in silico* pharmacology (Ekins et al., 2007), clinical big data (Mayo et al., 2017; Singh et al., 2018), *in vitro* pharmacology, and mathematical modeling (Dulong et al., 2015) to help expand our knowledge of the molecular clock and its relevance to human disease, consistent with the important trend toward personalized medicine. Here, we have analyzed and explored the case for chronotherapy as an option to improve the effectiveness of existing and future cytotoxic cancer therapies. A better appreciation for this underlying fundamental biological phenomenon and its potential for chronotherapy could benefit many cancer patients worldwide (**Figure 1**).

## Author Contributions

TK contributed the idea, the draft and conducted the interview with Professor Francis Lévi. AL contributed the overall structure and corrected the text.

## Funding

“European Union’s Horizon 2020 Research and Innovation Programme under the Marie Skłodowska-Curie grant agreement 675610” to TK.

## Conflict of Interest

AL is Co-Founder and CSO of Eisbach Bio GmbH. 

TK declares that the research was conducted in the absence of any commercial or financial relationships that could be construed as a potential conflict of interest.
