# Corrigendum: Exploiting the Circadian Clock for Improved Cancer Therapy: Perspective From a Cell Biologist

**DOI:** 10.3389/fgene.2020.00601

**Published:** 2020-06-19

**Authors:** Tia Tyrsett Kuo, Andreas G. Ladurner

**Affiliations:** ^1^Biomedical Center Munich, Faculty of Medicine, Ludwig-Maximilians-Universität München, Planegg-Martinsried, Germany; ^2^Max Planck Institute of Biochemistry, International Max Planck Research School for Molecular and Cellular Life Sciences, Martinsried, Germany; ^3^Center for Integrated Protein Science Munich (CIPSM), Ludwig-Maximilians-Universität München, Munich, Germany; ^4^Munich Cluster for Systems Neurology (SyNergy), Ludwig-Maximilians-Universität München, Munich, Germany

**Keywords:** chronotherapeutic, circadian rhythm, chemotherapy, cancer, precision (stratified) medicine

In the original article, we neglected to include the funder the “European Union's Horizon 2020 Research and Innovation Programme under the Marie Skłodowska-Curie grant agreement, 675610” to Tia Tyrsett Kuo.

The authors apologize for this error and state that this does not change the scientific conclusions of the article in any way. The original article has been updated.

